# Citrus Black Spot Symptoms on Fruit Exposed to *Phyllosticta citricarpa* Infections at Different Developmental Stages

**DOI:** 10.1002/pei3.70031

**Published:** 2025-01-26

**Authors:** Providence Moyo, Régis Oliveira Fialho, Geraldo José Silva Junior, Paul Fourie

**Affiliations:** ^1^ Citrus Research International Nelspruit South Africa; ^2^ Department of Plant Pathology and Nematology University of São Paulo Piracicaba São Paulo Brazil; ^3^ Department of Research and Development Fund for Citrus Protection (Fundecitrus) Araraquara São Paulo Brazil; ^4^ Department of Plant Pathology Stellenbosch University Stellenbosch South Africa

**Keywords:** disease incidence, fungicide sprays, inoculation, mandarin fruit, ontogenic resistance

## Abstract

Citrus black spot (CBS), caused by *Phyllosticta citricarpa*, is an important fungal disease of citrus. Higher CBS severity has been associated with *P. citricarpa* infections at the young and green stages of fruit. The length of the fruit susceptibility period may be influenced by the amount of inoculum and the climate of the citrus growing region. This study was conducted in orchards across two South African provinces to assess the intensity of CBS under two conditions: (i) unsprayed fruit of Valencia sweet orange, Nova, and Empress mandarins inoculated with *P. citricarpa* at 10^1^, 10^3^, and 10^5^ pycnidiospores/mL at various intervals after fruit set, from October (petal fall stage) to March (for mandarins) or June (for sweet orange), and (ii) non‐inoculated Valencia fruit from orchards subjected to spray programs ranging from one to nine applications between October and June. Higher CBS intensity was observed in Valencia fruit inoculated between November and January, with an average disease severity index (DSI) of 0.94, compared to a significantly lower (0.07) for fruit inoculated from February to June. Inoculations in November or December for Nova mandarins and in December for Empress with 10^5^ pycnidiospores/mL resulted in higher CBS intensity compared to those with lower pycnidiospore concentrations. The absence of fungicide applications from October to December during the 2017–2018 season led to increased CBS intensity, with a DSI of ~0.47, a value which was not significantly different from that of non‐treated control (NTC) trees. Leaving sweet orange trees unprotected from January onwards did not significantly increase the CBS intensity. These results confirm that citrus growers should focus on protecting citrus fruit, against *P. citricarpa* infection, in their dark green and young stages (c.15–30 mm diameter) to control CBS.

## 1 Introduction

Citrus black spot (CBS), caused by the fungus *Phyllosticta citricarpa* (McAlpine) van der Aa., affects the fruit rind without causing any internal decay. Most citrus species are affected by the disease, but lemons and sweet oranges are the most susceptible (Kiely 1948; Silva Junior et al. 2016; Miles et al. 2019). Other citrus species affected include grapefruit and mandarin (Wager 1952; Kiely 1948, 1969; Brodrick 1969; Kotzé 1981; Miles et al. 2019). Sour oranges ( *Citrus aurantium* ), along with their hybrids, Tahiti limes ( *C. latifolia* ), and pummelo ( *C. maxima* ), are generally regarded as the only citrus species resistant to CBS (Kotzé 1981; Miles et al. 2019). However, CBS symptoms have occasionally been observed on sour orange fruit in Brazil, although at very low severity levels (Silva Junior et al. 2016).

*Phyllosticta citricarpa* produces two types of spores: ascospores formed in pseudothecia on leaf litter on the ground in the orchard (Kiely 1948; McOnie 1964) and pycnidiospores (conidia) produced in pycnidia formed in leaf litter as well as in lesions on fruit, leaves and twigs (Kiely 1948; Kotzé 2000). The airborne ascospores are the primary inoculum of the pathogen. Pycnidiospores, which are dispersed by water downwards and at short distances, are considered to be of lesser importance in South Africa and Australia, compared with the aerially dispersed ascospores (Kiely 1948; McOnie 1964; Kotzé 1981), although they may contribute to in‐tree infections (Spósito et al. 2008, 2011; Carstens et al. 2017). Pycnidiospores have, however, been presumed to play a more significant role in CBS epidemics in high rainfall areas such as Brazil, Florida and Australia (Spósito et al. 2008, 2011; Wang et al. 2016; Hendricks, Christman, and Roberts 2017, 2020; Tran et al. 2020).

Symptomatology of CBS is characterized by a long incubation and latency period, in which most symptoms may not appear until fruit ripening. The intensity of the disease depends on several factors, which include the availability of inoculum, climatic conditions that are conducive to infection during the fruit susceptibility period as well as the age of fruit at the time of infection (Kotzé 1981, 2000). The critical period for infection extends from mid‐spring through summer (October to February in the Southern hemisphere) and hence, fungicide sprays for control of CBS are restricted to this period in South Africa, Argentina, and Australia (Kiely 1948; Kotzé 1981; McOnie 1964; Miles, Willingham, and Cooke 2004; Schutte, Beeton, and Kotzé 1997; Schutte et al. 2003; Fogliata et al. 2011).

The application of fungicides at 4‐ to 6‐week intervals during the critical period of infection has provided effective control of CBS in South Africa, and late season sprays (after January and onwards) are generally not necessary, regardless of the presence of inoculum and rainfall (Kiely 1948). However, when the South African control program was adopted for the control of CBS in São Paulo (SP), Brazil, similar levels of control were not achieved (Lanza et al. 2018). This was attributed to markedly more suitable conditions for CBS in Brazil, with higher volumes of rainfall and longer duration of the rainy period and generally higher inoculum potential. Further contributing factors include the presence of more than one flowering season and overlapping fruit sets triggered by the harvest after October of late‐maturing cultivars (Lanza et al. 2018). Moreover, the multiple blooms in association with high frequency of rain in areas with highly suitable climates for CBS, such as São Paulo and Ghana, result in fruit at various stages of susceptibility to infection and thus, extended periods of protection are required to control CBS in these regions (Spósito et al. 2008; Brentu et al. 2012; Lanza et al. 2018). In contrast, harvest occurs prior to bloom in September in South African and Australian citrus production, which is aimed at the fresh fruit market, and bloom periods are kept uniform with predominantly one fruit set per annual cycle (usually after fruit harvest), and fruit protection beyond February is rarely required (Kiely 1948; Kotze 1963; Kotzé 1981; McOnie 1964; Schutte, Beeton, and Kotzé 1997; Schutte et al. 2003; Miles, Willingham, and Cooke 2004).

Several studies have assessed the susceptibility of citrus fruit to CBS infection. Kotzé (1981) reported the critical period for infection being the 3 months after fruit set, and fungicide protection against CBS in South Africa and Australia are mostly required up to 150 days after petal fall (as reviewed in Guarnaccia et al. 2019). Aguiar et al. (2012); Frare et al. (2019); and Fialho et al. (2022) demonstrated that sweet orange fruit up to 7‐cm‐diameter were susceptible to artificial inoculation with *P. citricarpa* conidia. Lanza et al. (2018) and Moreira et al. (2023) highlighted the need for longer periods of fruit protection (up to 220 days) for late‐maturing cultivars and mature orchards in Brazil, but found no significant increase in disease incidence and severity from late season infections. Results from these studies, as well as Brentu et al. (2012) and Baldassari, Reis, and Goes (2006), indicated a trend of fruit becoming less susceptible with maturity, which suggests ontogenic resistance development. In addition, the lower CBS intensity on fruit inoculated at 7 cm diameter compared to smaller diameters may be associated with the long time needed (up to 360 days) from inoculation to CBS symptom appearance (Frare et al. 2019; Fialho et al. 2022). The aim of this study was, therefore, to assess the CBS symptom intensity on fruit of different ages, inoculated artificially with *P. citricarpa* or protected with fungicides, in South African orchards. Field studies examining fruit susceptibility at different developmental stages may provide critical insights for developing more effective and sustainable CBS management strategies.

## 2 Materials and Methods

### 2.1 Inoculation of *P. citricarpa* at Different Fruit Stages

The *P. citricarpa* isolate (PC.9), identified using quantitative polymerase chain reaction (Hu et al. 2014) and stored in the Citrus Research International (CRI) culture collection in Nelspruit, was obtained from a CBS lesion on Delta Valencia sweet orange fruit obtained from Malalane, Mpumalanga, and South Africa. Inoculum suspensions were prepared by flooding the surface of 14‐ to 21‐day‐old *P. citricarpa* cultures with sterile distilled water, scraping the surface and filtering conidia using a gauze pad, before concentrations were estimated with a hemocytometer and diluted to 10^1^, 10^3^, and 10^5^ pycndiospores/mL.

The study was conducted over a 5‐year period from 2017 to 2021 in sweet orange ( *C. sinensis* ) and mandarin ( *C. reticulata* ) commercial orchards. The trials on sweet orange were performed in an orchard located in Nelspruit, South Africa during the 2017–2018 and 2018–2019 seasons. This orchard was planted in 1988 and consisted of Valencia orange trees grafted onto Volkamer lemoniana (*C. volkameriana*), with spacing of 8 × 6 m. The trials on mandarin fruit were conducted during the 2019–2020 and 2020–2021 seasons in orchards located in the North–West province of South Africa. One trial conducted in Mooinooi consisted of Nova mandarin grafted onto Carrizo citrange ( *C. sinensis* × *Poncirus trifoliata* ), which was planted in 1998 with a tree spacing of 6 × 2 m. The second trial was conducted in a Brits orchard planted in 2011, consisting of Empress mandarin grafted onto Swingle citrumelo ( *C. sinensis* × *P. trifoliata* × *C. paradisi* ), also spaced 6 × 2 m. No fungicides were sprayed on trees used for these experiments.

The treatments were arranged in a completely randomized design with 30 treatments with five replicates for Valencia sweet orange (9 inoculation times × 3 spore concentrations + 3 positive controls) and 21 treatments with five replicates for Nova and Empress mandarins (6 inoculation times × 3 spore concentrations + 3 positive controls). The treatments consisted of fruit inoculation either 10 or 7 times, using three spore concentrations. The three positive control treatments consisted of fruit inoculated with each spore concentration across multiple months, resulting in a total of 10 inoculations per fruit for Valencia sweet orange and 7 inoculations per fruit for Nova and Empress mandarins.

Fruit were inoculated monthly with a *P. citricarpa* pycnidiospore suspension, from October until March (early maturing mandarins that are harvested between April and June) or June (late‐maturing sweet orange that are harvested from end of July to August/early September). At each inoculation date (a given month) and for each spore concentration (10^1^, 10^3^, and 10^5^ pycnidiospores/mL), four fruit were each sprayed with approximately 10 mL of pycnidiospore suspension, using a hand‐held bottle sprayer (Fialho et al. 2022). All three concentrations were applied on one tree and replicated five times in different trees located in five different rows. All inoculated fruit were clearly marked but not removed from the trees. After inoculation, fruit were kept in small humid chambers (transparent plastic bags) for ~48 h. Non‐inoculated fruit were marked and used as controls. Fruit diameter and color were measured on 20 fruits randomly collected at each inoculation date. The fruit diameter was measured using an electronic caliper (CD‐6″ C, Mitutoyo Corp, Tokyo, Japan), whereas fruit color was visually assessed using the No. 34 and No. 36 CRI color charts for oranges and mandarins, respectively. The color T8 was defined as dark green and T1 a fully developed color (Citrus Research International 2004a, 2004b).

### 2.2 Fungicide Sprays for CBS Control at Different Fruit Stages

Sweet orange trees were subjected to a staggered fungicide spray program exposing fruit to different periods of natural infection by the CBS pathogen, between the 2017 and 2020 growing seasons (Table 1). Trials were conducted in two Valencia orchards grafted onto rough lemon ( *C. jambhiri* ). The first orchard, planted in 1986 with a spacing of 8.3 × 5.6 m, was used during the 2017–2018 season, whereas the second orchard, planted in 1982 with a spacing of 8.3 × 5.5 m, was utilized in the 2018–2019 and 2019–2020 seasons.

**TABLE 1:** Spray programs consisting of mancozeb (Mz) and copper oxychloride (Cu) applied in alternation and exposing Valencia sweet orange fruit to different periods of natural infection by *Phyllosticta citricarpa*, during three seasons (2017/2018, 2018/2019, and 2019/2020), in Mpumalanga province of South Africa.

2017/2018 season									
Number of sprays	CBS control program								
	0^a^	24	49	84	109	134	169	192	217
	0^b^	24	25	35	25	25	35	23	25
	16‐October^c^	09‐November	4‐December	08‐January	2‐February	27‐March	3‐April	26‐April	21‐May
0	Non‐treated trees								
1	Mz								
2	Mz	Mz							
3	Mz	Mz	Cu						
4	Mz	Mz	Cu	Mz					
5	Mz	Mz	Cu	Mz	Mz				
6	Mz	Mz	Cu	Mz	Mz	Cu			
7	Mz	Mz	Cu	Mz	Mz	Cu	Mz		
8	Mz	Mz	Cu	Mz	Mz	Cu	Mz	Mz	
9	Mz	Mz	Cu	Mz	Mz	Cu	Mz	Mz	Cu

2018/2019 season									
Number of sprays	0	24	45	80	104	129	164	189	214
	0	24	21	35	24	25	35	25	25
	12‐October	05‐November	26‐November	31‐December	24‐January	18‐February	25‐March	19‐April	14‐May
0	Non‐treated trees								
1	Mz								
2	Mz	Mz							
3	Mz	Mz	Cu						
4	Mz	Mz	Cu	Mz					
5	Mz	Mz	Cu	Mz	Mz				
6	Mz	Mz	Cu	Mz	Mz	Cu			
7	Mz	Mz	Cu	Mz	Mz	Cu	Mz		
8	Mz	Mz	Cu	Mz	Mz	Cu	Mz	Mz	
9	Mz	Mz	Cu	Mz	Mz	Cu	Mz	Mz	Cu

2019/2020 season									
Number of sprays	0	24	49	85	109	135	169	193	218
	0	24	25	36	24	26	34	24	25
	14‐October	07‐November	2‐December	07‐January	31‐January	26‐February	31‐March	24‐April	19‐May
0	Non‐treated trees								
1	Mz								
2	Mz	Mz							
3	Mz	Mz	Cu						
4	Mz	Mz	Cu	Mz					
5	Mz	Mz	Cu	Mz	Mz				
6	Mz	Mz	Cu	Mz	Mz	Cu			
7	Mz	Mz	Cu	Mz	Mz	Cu	Mz		
8	Mz	Mz	Cu	Mz	Mz	Cu	Mz	Mz	
9	Mz	Mz	Cu	Mz	Mz	Cu	Mz	Mz	Cu

^a^ Number of days the fruit were kept protected by fungicides.

^b^ Number of days between sprays.

^c^ Spray dates; Mz, mancozeb applied at 200 g/100 L of water; Cu, copper oxychloride applied at 200 g/100 L of water.

The spray program consisted of contact fungicides that are effectively used in a spray program to control CBS in South Africa, that is, mancozeb (Dithane M‐45800 WP, 80% of mancozeb, Dow AgroSciences, Bryanston, South Africa) applied in alternation with copper oxychloride (Copper Oxychloride WP, 50% metallic copper, Universal Crop Protection, Kempton Park, South Africa). Both mancozeb and copper oxychloride were applied as per their registered rates, that is, 200 g of each fungicide per 100 L of water. Trees were sprayed to runoff using a trailer‐mounted, high‐volume, and high‐pressure (2500–3000 kPa) sprayer with two hand‐held spray guns.

The trial consisted of 10 treatments, in which mancozeb was applied twice, at 25‐day intervals, followed by a copper spray applied at a 35‐day interval before the next mancozeb application (Table 1). The sprays began in October (80% petal fall) of each growing season. All data trees were sprayed with mancozeb, except for five trees that were left unsprayed for the duration of the trials to serve as non‐treated controls (NTC). The second fungicide sprays were applied in November, where all data trees treated with mancozeb in October were treated except for one set of five trees, which was left untreated. During the third fungicide sprays conducted in December, all trees were treated with copper oxychloride except for two sets of five trees, that is, the set of trees left untreated in November plus an additional set of five trees (Table 1). This continued monthly such that at the end of the trial, sets of five trees received from 0 to 9 fungicide sprays. Each treatment was replicated five times, in which an individual tree represented a replicate. A total of nine spray dates were recorded within a period of up to 218 days.

### 2.3 Weather Data

Environmental conditions including rainfall, temperature, and relative humidity were recorded using an iLeaf weather station (Hortec, Somerset West, South Africa) located ~6 km from the Valencia orchards where trials were conducted. No weather station was available at both trial sites in the North–West province.

### 2.4 Disease Evaluation and Statistical Analysis

Disease assessments on fruit were performed 1 week before harvest in September for oranges, and in April and May of each season for Nova and Empress mandarin, respectively. All inoculated fruit in the inoculation trials (20 fruit per inoculation month and spore concentration combination) and 500 fruit (100 fruit per data tree) corresponding to each different set of five trees in the staggered spray trials, were rated for CBS intensity. CBS intensity was assessed using three classes of lesions: class 0 = fruit without lesions, class 1 = fruit with one to three lesions, and class 2 = fruit with four or more lesions (Schutte, Beeton, and Kotzé 1997; Schutte et al. 2003). The disease severity was expressed as disease severity index (DSI) on a proportion basis according to Chiang, Liu, and Bock (2017). The formula for a DSI was written as follows:

DSI = [(no. of fruit without lesions × 0) + (no. of fruit with one to three lesions × 1) + (fruit with four or more lesions × 2)]/(total number of fruit × maximal disease index). The numerator represents the sum of the number of fruit recorded per class per tree (ranging from 0 to 4 for inoculation trials and 0 to 100 for spray trials) multiplied by its respective lesion class (0 to 2). The denominator represents the total number of fruit assessed per tree (4 for inoculation trials and 100 for spray trials) multiplied by the maximum disease index (class 2).

Blanca et al. (2017) tested the robustness of the *F*‐test (ANOVA) under a wide variety of conditions involving non‐normal distributions and found it to be a reliable and robust statistical procedure for use on data deviating from normality. Therefore, although our data and transformed data were typically variable and not normally distributed, CBS intensity assessed in both the inoculation and staggered spray trials were subjected to one‐way analysis of variance (ANOVA). When significant differences were detected by the *F*‐test (*p* < 0.05), means were compared using Tukey's test at a 5% significance level. ANOVA was performed using software R v. 3.6.1 (R Core Team 2019) with the add‐on package “ExpDes.”

## 3 Results

### 3.1 Inoculation of *P. citricarpa* at Different Fruit Stages

**FIGURE 1:**
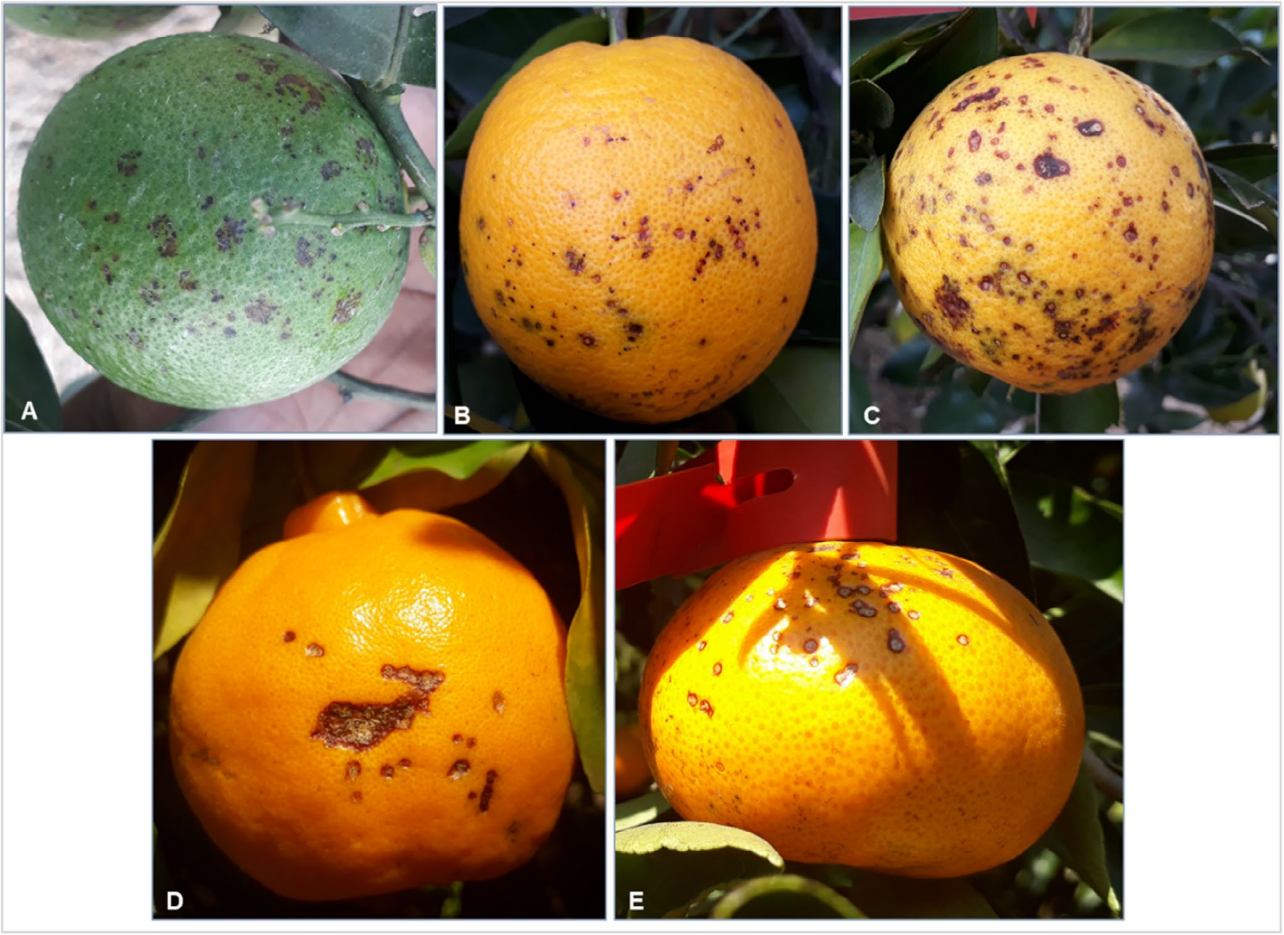
Citrus black symptoms observed on sweet orange and mandarin fruit artificially inoculated with pycnidiospore suspensions of *Phyllosticta citricarpa*. (A) False melanose symptoms appearing as small dark spots on the rind of green Valencia sweet orange fruit. (B) Freckle spot on Valencia fruit. (C) Virulent spots on Valencia fruit. (D) Virulent spots on Empress mandarin fruit. (E) Hard spots on Nova mandarin fruit.

Inoculation of sweet orange and mandarin fruit with pycnidiospore suspensions of *P. citricarpa* resulted in characteristic symptoms of CBS, confirming the effectiveness and reproducibility of the method used. Symptoms observed included freckle spot, hard spot, virulent spots and false melanose (Figure 1). False melanose symptoms appeared as small black spots on green fruit (Figure 1A), whereas freckle spot appeared as deep orange to brick red lesions (Figure 1B). Virulent spots (Figure 1C,D) were often observed on fruit inoculated with the highest spore concentration, and hard spot lesions contained pycnidia at the centre (Figure 1E). Non‐inoculated fruit did not display any symptoms, and therefore the data were not included in the analyses.

#### 3.1.1 Inoculation of Valencia Orange Fruit

**FIGURE 2:**
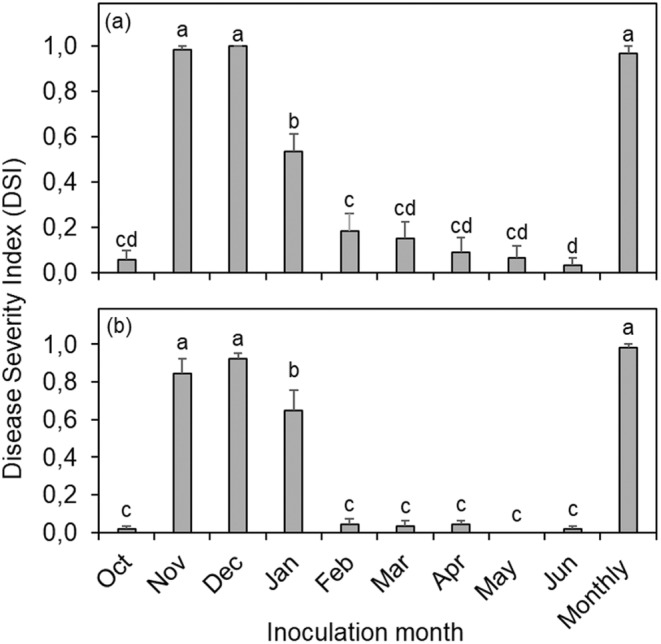
Citrus black spot intensity, expressed as disease severity index (DSI), assessed 1 week before harvest of Valencia sweet orange orchard located in the Nelspruit area, South Africa, during the 2017–2018 (a) and 2018–2019 (b) seasons. Columns represent pooled data of fruit inoculated with 10^1^, 10^3^, or 10^5^ pycnidiospores/mL of *Phyllosticta citricarpa* once a month from October to June. Monthly refers to fruit inoculated nine times, once a month, from October to June. Columns followed by the same letters do not differ statistically by Tukey test (*p < 0.05*). Bars indicate the standard error of the mean.

The ANOVA for the DSI revealed a significant interaction between treatments (month of inoculation) and seasons (*p <* 0.001) (Figure 2). However, no significant differences were observed in the DSI of CBS across the three spore concentrations (*p =* 0.141). Similar CBS symptom expression trends were observed between the two seasons. High intensity of CBS symptoms was observed on fruit inoculated in November and December, moderate for inoculations in January, and low for fruit inoculated in the other months. Fruit inoculated in December showed the highest intensity of CBS, with a DSI of 1.00 and 0.92 in 2017–2018 (Figure 2a) and 2018–2019 (Figure 2b), respectively. No significant differences in the DSI were found among fruit inoculated in November, December and monthly, regardless of the season (Figure 2a,b). DSI was higher than 0.96 when fruit were inoculated with *P. citricarpa* in November, December, and monthly, whereas it reached 0.54 and 0.64 for the January inoculations in the two seasons (Figure 2).

Significantly more fruit remained asymptomatic following inoculations in October and from February to June (results not shown). The DSI of fruit inoculated in October was 0.06 and 0.017 for the 2017–2018 and 2018–2019 seasons, respectively. Fruit inoculated from February to June also showed a lower DSI with values ranging from 0.19 to 0.033 for the 2017–2018 season and 0 to 0.043 for the 2018–2019 season. The intensity of CBS during each of these months was not significantly different from each other, regardless of the season (Figure 2).

**FIGURE 3:**
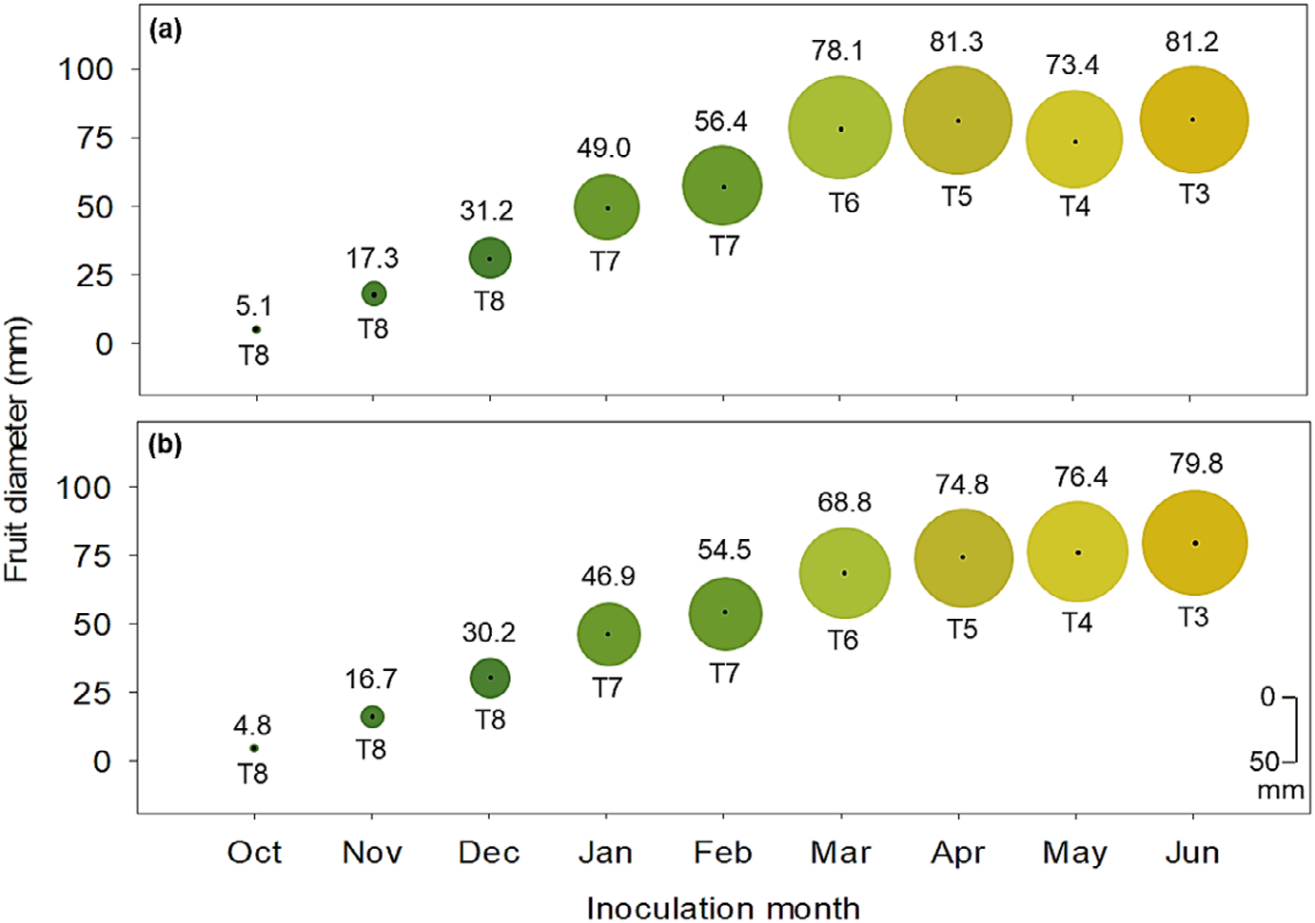
Average diameter (value in mm above circle) and color indices (T8–T3) of Valencia sweet orange fruit (below circle) at each month of inoculation with pycnidiospore suspensions of *Phyllosticta citricarpa*, during the 2017–2018 (a) and 2018–2019 (b) seasons, in the Nelspruit area, South Africa. Color of fruit rind at each inoculation month was determined using the No. 34 Citrus Research International (CRI) color charts for oranges.

The average diameter of inoculated fruit ranged between 5.1–81.2 mm and 4.8–79.8 mm in 2017–2018 and 2018–2019, respectively (Figure 3). Fruit were dark green (T8) at the onset of inoculations in October, then the color index changed at each inoculation from T8 until reaching T3 (light orange) in the last inoculation in June (Figure 3).

#### 3.1.2 Inoculations of Mandarin Fruit

**FIGURE 4:**
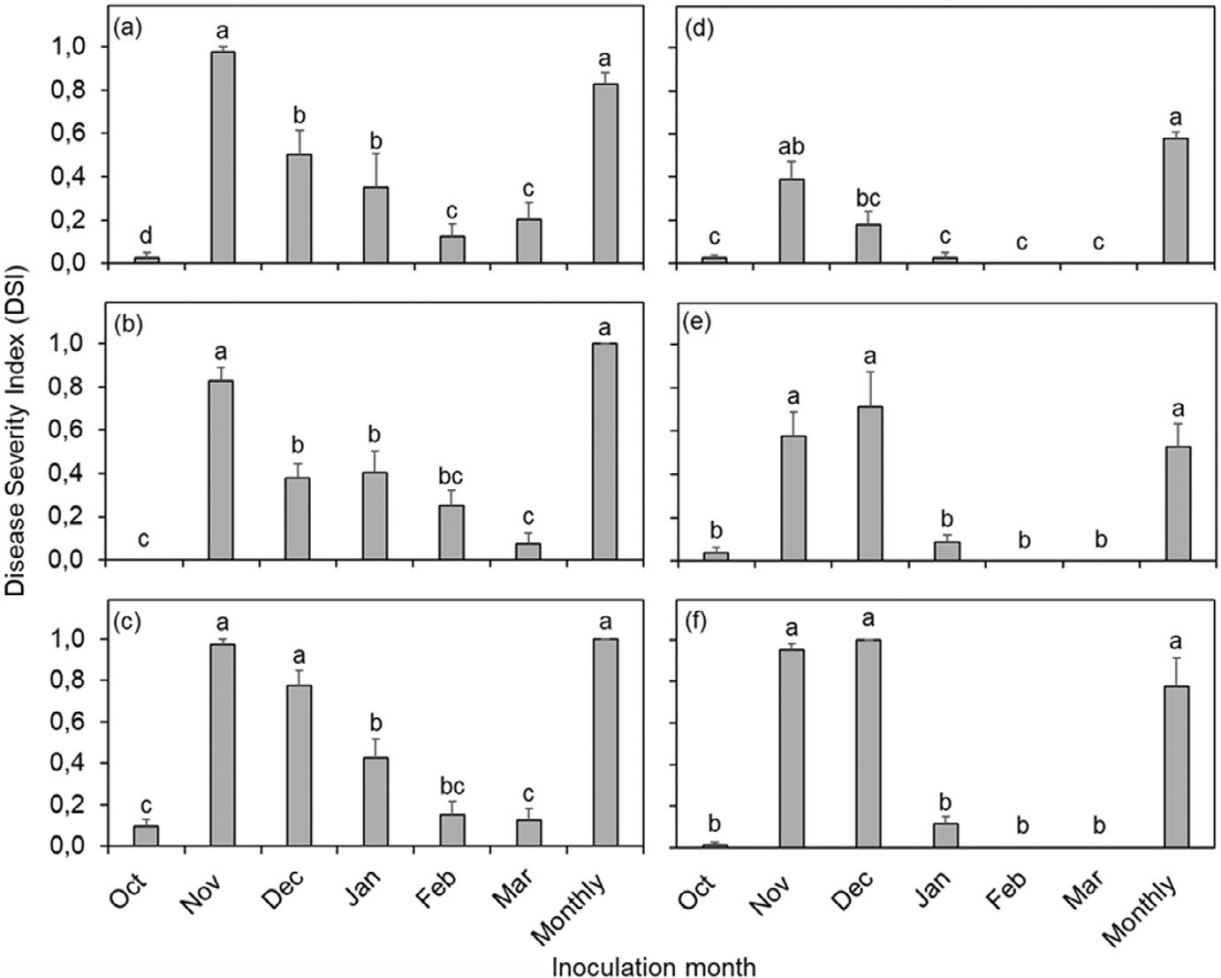
Citrus black spot intensity expressed as Disease Severity Index (DSI) assessed 1 week before harvest of the Nova mandarin orchard located in the Mooinooi area, South Africa, during the 2019–2020 (a–c) and 2020–2021 (d–f) seasons. Columns represent pooled data of fruit inoculated with 10^1^ (a, d), 10^3^ (b, e), or 10^5^ (c, f) pycnidiospores/mL of *Phyllosticta citricarpa* once a month from October to March. Monthly refers to fruit inoculated six times, once a month, from October to March. Columns followed by the same letters do not differ statistically by Tukey test (*p < 0.05*). Bars indicate the standard error of the mean.

The ANOVA for CBS intensity on Nova mandarin fruit showed a significant interaction among seasons × treatment (month of inoculation) × pycnidiospore concentration (*p* < 0.0001) (Figure 4). In the 2019–2020 season (Figure 4a–c), DSI of Nova mandarin fruit inoculated in November with 10^1^ (Figure 4a), 10^3^ (Figure 4b), and 10^5^ (Figure 4c) pycnidiospores/mL was 0.98, 0.83, and 0.98, respectively. The above‐mentioned CBS intensity was not significantly different from that of fruit inoculated monthly (DSI over 0.8) in both seasons. Fruit inoculated only in December with 10^5^ pycnidiospores/mL (Figure 4c) resulted in DSI value of 0.78, which did not differ from the DSI value of 1.00 obtained for fruit inoculated monthly (Figure 4c). Overall, fruit inoculated in October, February, or March exhibited lower CBS intensities, with DSI values below 0.30, irrespective of the pycnidiospore concentration used. In the 2020–2021 season, except for fruit inoculated in November or December with 10^5^ pycnidiospores/mL, the disease intensity was slightly lower in all the other months compared to the previous season (Figure 4). The DSI values ranged from 0.58 to 1.00 when the fruit was inoculated only in November or December with 10^3^ (Figure 4e) or 10^5^ (Figure 4f) pycnidiospores/mL, and no significant differences were observed between them and the fruit inoculated on a monthly basis. Similar to the 2019–2020 season, fruit inoculated in October, January, February, or March exhibited lower disease intensity, compared to that inoculated in November, with DSI values peaking at 0.10 only for those inoculated in January with 10^5^ pycnidiospores/mL (Figure 4f).

**FIGURE 5:**
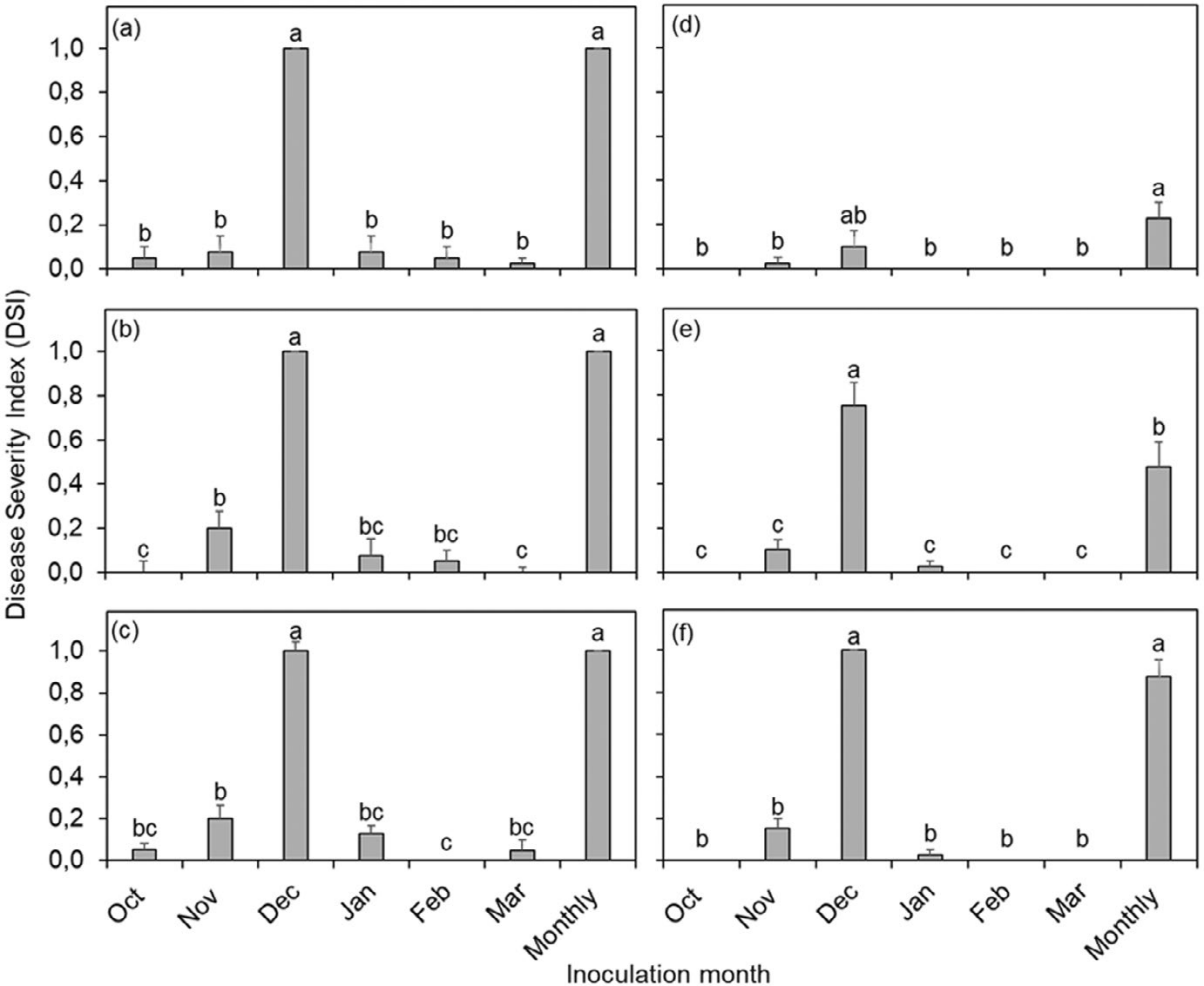
Citrus black spot intensity expressed as disease severity index (DSI) assessed 1 week before harvest of Empress mandarin orchards located in the Brits area, South Africa, during the 2019–2020 (a–c) and 2020–2021 (d–f) seasons. Columns represent pooled data of fruit inoculated with 10^1^ (a, d) 10^3^ (b, e), or 10^5^ (c, f) pycnidiospores/mL of *Phyllosticta citricarpa* once a month from October to March. Monthly refers to fruit inoculated six times, once a month, from October to March. Columns followed by the same letters do not differ statistically by Tukey test (*p* < 0.05). Bars indicate the standard error of the mean.

A significant interaction (*p* < 0.0001) among treatments of inoculation month, inoculum concentration and seasons was found for CBS intensity on Empress mandarin fruit (Figure 5). Across both seasons, CBS intensity was strongly influenced by the month of inoculation. In the 2019–2020 season, Empress fruit inoculated in December or on a monthly basis reached a DSI of 1.00, regardless of pycnidiospore concentration, and their CBS intensity differed significantly from the other months, but no significant differences were observed between them (Figure 5a–c). Conversely, Empress fruit inoculated only in October, November, January, February or March resulted in DSI values ranging from 0 to 0.20, regardless of pycnidiospore concentration (Figure 5a–c). A similar trend was observed in the 2020–2021 season with significantly higher DSI values observed on Empress fruit inoculated with 10^5^ pycnidiospores/mL in December or on a monthly basis, compared to the other months (Figure 5d–f). Fruit inoculated with 10^5^ pycnidiospores/mL in December or monthly inoculations reached peak DSI values of 1.00 and 0.88, respectively, whereas fruit inoculated in other months showed a maximum DSI of 0.15 (November) (Figure 5d–f).

**FIGURE 6:**
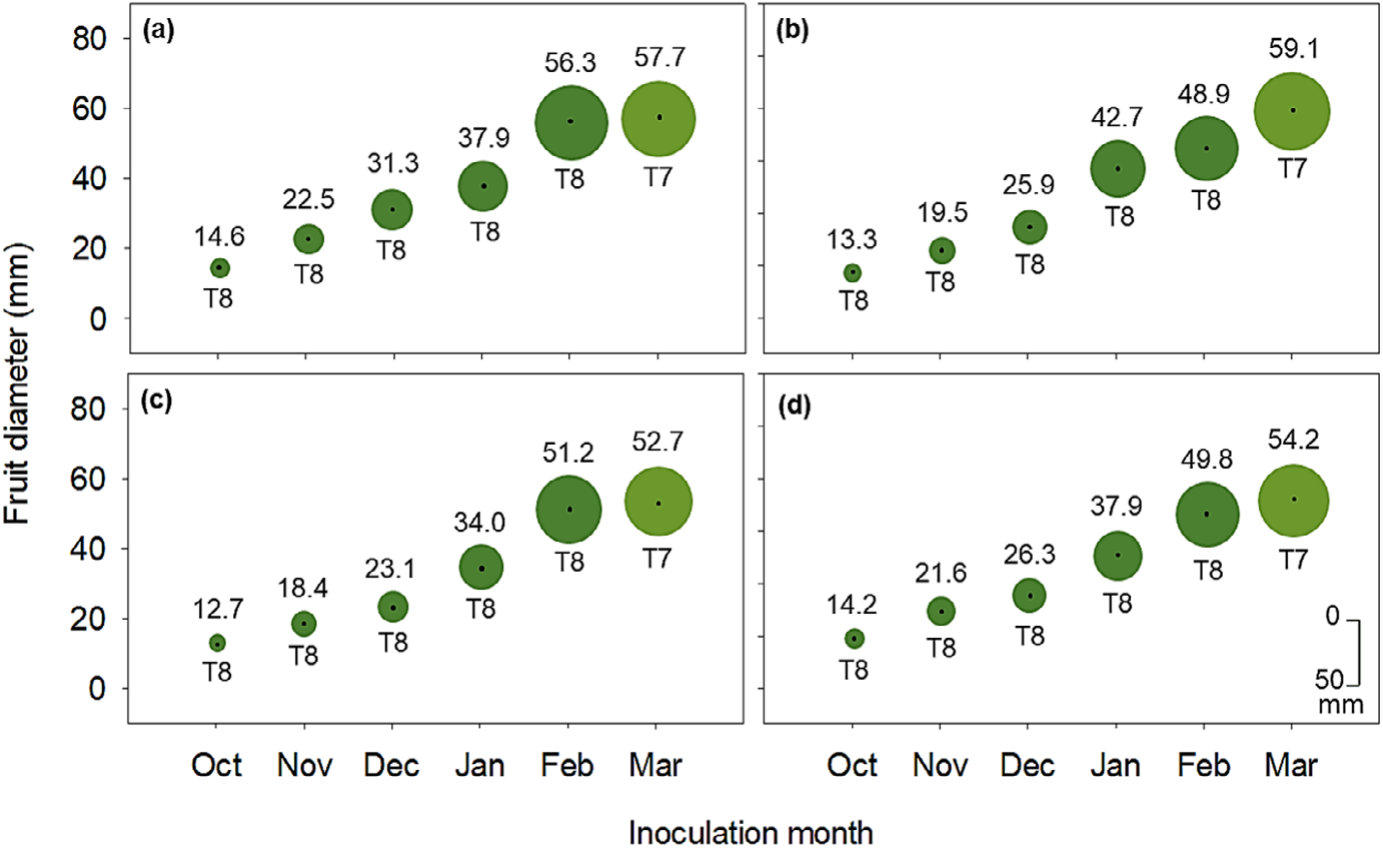
Average diameter (value above circle) and color of Nova and Empress mandarin fruit (below circle) at each month of inoculation with pycnidiospore suspensions of *Phyllosticta citricarpa*, during the 2019–2020 (a, c) and 2020–2021 (b, d) seasons, in Mooinooi and Brits, South Africa, respectively. Color of fruit rind at each inoculation month was determined using the No. 36 Citrus Research International (CRI) color chart for mandarins.

For both seasons, Nova mandarin fruit were dark green (T8) at the onset of inoculations in October and the color index stayed unchanged during each inoculation until the last inoculation which was conducted in March when the color index was T7 (Figure 6a,b). Average fruit diameter ranged from 14.6 to 57.7 mm and 13.3 to 59.1 mm in the first and second season, respectively (Figure 6a,b). Inoculations on Empress fruit were mostly conducted on fruit exhibiting the T8 color index but fruit exhibited the T7 color index during the last inoculations in March (Figure 6c,d).

### 3.2 Fungicide Sprays for CBS Control at Different Fruit Stages

**FIGURE 7:**
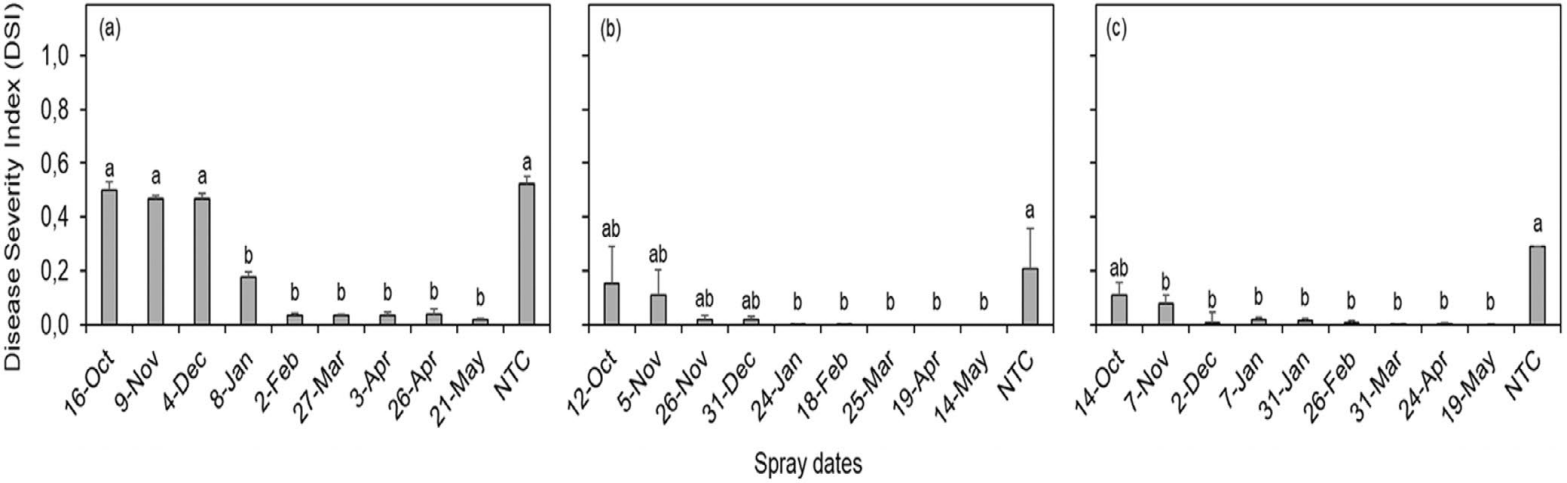
Citrus black spot intensity expressed as disease severity index (DSI) from trees treated with one to nine fungicide sprays (mancozeb and copper oxychloride), during the 2017–2018 (a), 2018–2019 (b) and 2019–2020 (c) seasons, in the Mpumalanga province of South Africa. NTC, non‐treated control trees. Columns followed by the same letters do not differ statistically by Tukey test (*p* < 0.05). Bars indicate the standard error of the mean.

The data for the different seasons were analyzed separately as the CBS intensity during the 2017–2018 (Figure 7a) season was significantly higher (*p* < 0.05) compared to that observed in the 2018–2019 (Figure 7b) and 2019–2020 (Figure 7c) seasons. CBS incidence on non‐treated trees (NTC) during the 2017–2018 season reached a DSI of 0.52 in comparison to 0.21 and 0.29 in the two following seasons, respectively (Figure 7). A clear trend of a decrease in the intensity of CBS with an increase in the number of fungicide sprays and fruit maturation was evident in the 2017–2018 season, but not very clear during the last two seasons due to the low CBS pressure experienced (Figure 7b,c). The application of four or more fungicide sprays (protecting fruit from October to January, and/or onwards) significantly reduced the CBS intensity when compared to NTC in the 2017–2018 season. Fruit protected only from October to December during the 2017–2018 and 2018–2019 seasons showed average DSI values of approximately 0.50 and 0.07, respectively, with no significant differences compared to NTC (Figure 7a,b). During the 2018–2019 and 2019–2020 seasons, CBS intensity was notably low, resulting in no significant differences in DSI values among treated fruit. Moreover, fruit that were sprayed between one and four times in the 2018–2019 season and just once (October) in 2019–2020 did not differ significantly from NTC (Figure 7b,c).

#### 3.2.1 Weather Data

**FIGURE 8:**
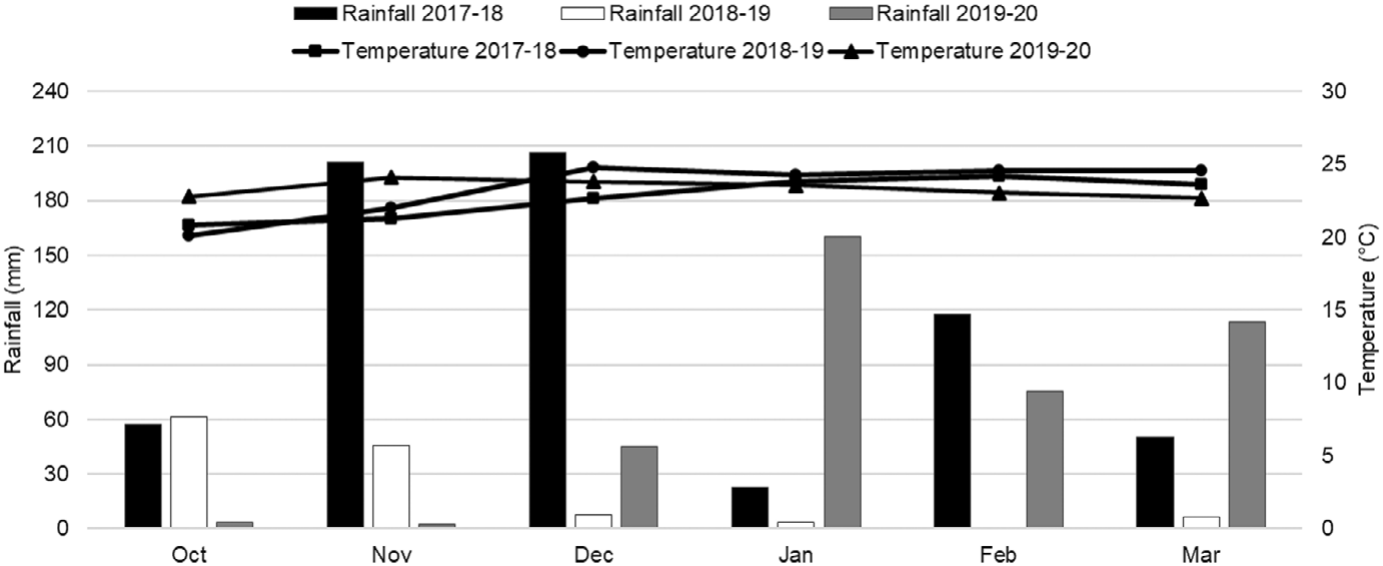
Monthly rainfall and average temperatures during the fungicide application periods for citrus black spot control on Valencia orange orchards in the 2017–2018, 2018–2019, and 2020–2021 seasons in Nelspruit, South Africa.

Rain events (≥ 0.3 mm) were recorded during the period from October to March for all three seasons in the Nelspruit area where trials were conducted on Valencia sweet orange orchards (Figure 8). The highest rainfall was recorded in the 2017–2018 season, which was also the season with the most incidence of diseased fruit on untreated trees. Seventy‐five rainy days and a total of 655.20 mm of rain was recorded during the 2017–2018 season, with most rainfall occurring during the months of November and December (Figure 8). A total of 124.8 mm and 399.24 mm rain was recorded during the 2018–2019 (40 rainy days) and 2019–2020 seasons (82 rainy days), respectively. The mean temperatures during this period were 22.73°C, 23.4°C, and 23.33°C for the 2017–2018, 2018–2019, and 2019–2020 seasons, respectively.

## 4 Discussion

This study demonstrated the period of susceptibility of sweet oranges and mandarins to *Phyllosticta citricarpa* in South Africa by using unsprayed and inoculated trees as well as sweet orange trees treated with different fungicide applications for CBS control and infected by natural inoculum. Our results showed that fruit of the two citrus species were more susceptible when inoculated at green and small stages from November to January. Expression of CBS symptoms associated with inoculations in young fruit stages was significantly more pronounced than in the artificial inoculations from February onwards, when the fruit were more than 50 mm (sweet oranges) and 23 mm (mandarins) in diameter. The absence of fungicide applications from October to December led to increased CBS intensity, whereas applications performed from October to January onward did not further reduce the already low CBS intensity. Therefore, our findings indicate that the critical period for protecting fruit against CBS occurs during the early stages of fruit development.

The observations on Valencia sweet orange fruit agree with results of Frare et al. (2019) who reported higher incidences of CBS on 15 mm diameter Hamlin, Pera and Valencia sweet orange fruit grown in a greenhouse and lower values when diameter was 50 or 70 mm. Fialho et al. (2022) also observed high CBS incidence and severity on sweet orange fruit inoculated in November (~15 mm diameter) and December (~30 mm) in commercial orchards grown in the São Paulo citrus belt, Brazil. As with the current study, inoculation of ripening fruit performed by Fialho et al. (2022) resulted in lower CBS incidence and a consistent decrease in CBS intensity on Valencia fruit inoculated when fruit diameter was > 58 mm from March onwards.

Results on Nova mandarin fruit varied between seasons. In the first season (2019–2020), CBS levels declined significantly between November and March. With regards to the lower concentrations, a peak DSI value of 1.00 was recorded in November on fruit inoculated with 10^1^ pycnidiospores/mL, whereas in the following season, a DSI peak was recorded during December (0.74) on fruit inoculated with 10^3^ pycnidiospores/mL. Infection levels declined significantly in January with no symptoms observed in February and March in the 2020–2021 season. Fruit inoculated with 10^5^ pycnidiospores/mL in November or December had the highest DSI levels across both seasons. The statistical analysis revealed differences in CBS intensity on Empress mandarin fruit based on pycnidiospore concentration. High and severe infection levels across all three concentrations were only observed in fruit inoculated in December, suggesting a significantly shorter period of fruit susceptibility. The reasons for this variance in the results for mandarins are unclear, and further work will be required to determine the critical period of susceptibility and the factors involved in fruit infection. Although the inoculation protocol in this study effectively ensured adequate inoculum and suitable conditions for *P. citricarpa* infection throughout the trials, no weather station data were available for the mandarin trials. Whether the observations made could partly be attributed to environmental conditions not being conducive for symptom expression during the rest of the season, during the two seasons, or the differences in susceptibility to the pathogen by the mandarin types remain to be proven.

The intensity of CBS symptoms was often higher with the highest pycnidiospore concentration in our study, particularly on Nova fruit inoculated during the November or December, and on Empress fruit inoculated in December. No significant differences were detected in the DSI on Valencia fruit inoculated with the three pycnidiospore concentrations. The effect of pycnidiospore concentration on CBS intensity was also observed by Frare et al. (2019) and Fialho et al. (2022) who reported that sweet orange fruit inoculated with higher pycnidiospore concentrations displayed increased CBS intensity in Brazil. Furthermore, Tran et al. (2018) reported that Troyer citrange leaves and Murcott tangor fruit inoculated with a higher concentration (10^5^ spores/mL) of ascospores and pycnidiospores of *P. citricarpa* had more CBS symptoms than those inoculated with 10^4^ spores/mL.

The weather conditions in the Nelspruit area were most conducive for CBS occurrence during the 2017–2018 season and hence, the highest intensity of diseased fruit on untreated Valencia trees was recorded during this season compared to the other seasons. A substantial amount of rainfall was recorded from January until March (~200 mm), but the most rainfall occurred during November and December (~410 mm) of this season, which are also the months when fruit is reported to be most susceptible to CBS infection (Kiely 1948; McOnie 1964; Kotzé 1981; Schutte, Beeton, and Kotzé 1997; Schutte et al. 2003; Miles et al. 2008; Fogliata et al. 2011; Fialho et al. 2022). The lowest amount of rainfall was recorded during the 2018–2019 season and thus, the incidence of diseased fruit on untreated trees was lowest during this season.

Protecting Valencia orange fruit with fungicides during different periods after petal fall revealed that there is no need for extended sprays for CBS control in South Africa after January/February. The absence of fungicide sprays from October to December resulted in greater CBS intensity, whereas sprays from October to January onwards did not result in significant decrease in CBS disease severity index. This agrees with previous studies that report that the critical susceptibility period of citrus fruit to *P. citricarpa* infection extends from October to January/February in South Africa, Argentina and Australia and that fungicide sprays against the CBS pathogen are not necessary after this period (Kiely 1948; McOnie 1964; Kotzé 1981; Schutte, Beeton, and Kotzé 1997; Schutte et al. 2003; Miles et al. 2008; Fogliata et al. 2011). This critical period for *P. citricarpa* infection corresponds to 120–150 days of CBS suitable conditions after petal fall.

The CBS control programs in South Africa are shorter compared to the 180–220 days of fruit protection recommended for São Paulo (Silva Junior et al. 2016; Lanza et al. 2018; Moreira et al. 2023). This could partly be attributed to fewer conducive conditions for CBS outbreaks from February to April in South Africa (Schutte et al. 2003; Fourie et al. 2013). Furthermore, Valencia fruit is harvested around August to September in South Africa, whereas in Brazil, Valencia fruit is harvested until December (Lanza et al. 2018; Moreira et al. 2023). The results from the current study as well as those of Frare et al. (2019) and Fialho et al. (2022), show low susceptibility levels of citrus fruit to the CBS pathogen from February/March onwards. Whether these observations are due to the less conducive conditions for CBS development, the short incubation period from infection to the time when symptoms may be expressed or the indication of fruit developing progressive resistance to infection is not clear. The phenomenon of plant tissues becoming more resistant to infection as they mature has also been documented in other citrus diseases. For instance, fruit remain susceptible to the melanose fungus, *Diaporthe citri*, for 12 weeks after petal fall (Whiteside 1980), and are susceptible to *Alternaria alternata* (cause of Alternaria brown spot) from petal fall until they reach about 5 cm in diameter (Timmer et al. 2000). Similarly, fruit of 20–40 cm in diameter have been found to be more susceptible to the citrus canker pathogen, *Xanthomonas citri* subsp. c*itri* (Graham et al. 1992).

The phenomenon in which maturing plants or plant organs become less susceptible to pathogens has been observed in many plant‐pathogen systems and has been given several names including ontogenic resistance and age‐related resistance (Panter and Jones 2002; Whalen 2005; Develey‐Rivière and Galiana 2007). The findings of our study certainly suggest ontogenic resistance development of citrus fruit developing progressive resistance to *P. citricarpa* infection, but confirmation needs more assays focussed on the infection process through histopathological studies. Moreover, there has been little success in identifying sources of resistance to CBS, and even less in incorporating resistance into commercial citrus cultivars.

The ontogenic resistance to fungal infection by plants may be related to several factors, both chemical and morphological (Ficke et al. 2004). Ontogenic resistance in lime leaves ( *C. aurantifolia* ) to withertip disease, caused by *Colletotrichum limetticola*, has been linked to the change in composition of the cuticular membrane that occurs as lime leaves age (Roberts and Martin 1963). Three potential mechanisms that contribute to ontogenic resistance and help defend against pathogens following cuticle penetration have been identified in older apple leaves. These mechanisms involve (i) a reduction in cellular tissue pH, (ii) the inactivation of enzymes that degrade the cell wall, and (iii) the production of antimicrobial metabolites (MacHardy 1996).

The possible mechanisms of resistance by citrus plants to CBS are currently unknown. Histopathology experiments may reveal whether the observed lower susceptibility levels of citrus fruit to *P. citricarpa* after January are due to the onset of ontogenic resistance or not. Moreover, ontogenic resistance to *P. citricarpa* in citrus fruit may not necessarily indicate complete resistance (Ficke et al. 2002), but it may affect both infection and disease progress. The age‐related resistance may also contribute to reducing the required period of fungicide protection, as high levels of CBS fruit infection will require the presence of younger and more susceptible fruit stages, suitable weather conditions, and availability of inoculum in the orchard.

Overall, results from this study indicate the period from November to January as the most critical period for *P. citricarpa* infection in South Africa, confirming earlier reports (Kotzé 1981). This period corresponds to the period in which CBS fungicide sprays have traditionally been recommended, and application of fungicides within this period has proved to be sufficient to control CBS. Exceptions may occur in situations of high inoculum pressure, in which growers may consider adding sprays, particularly for later maturing cultivars. Therefore, this study may help to optimize CBS management by using a more sustainable fungicide spray program only during the period of fruit susceptibility.

## Conflicts of Interest

The authors declare no conflicts of interest.

## Data Availability

The data obtained in the study are presented in the article.
